# Supplementary material for: Secondary Structure of Subgenomic RNA M of SARS-CoV-2
Source: Viruses. 2022 Feb 4;14(2):322. doi: 10.3390/v14020322 (PMC8878378; doi:10.3390/v14020322)
Supplement: Supplementary file 1 [file viruses-14-00322-s001.zip › Supporting Information_1.pdf]

Supporting Information

Secondary structure of subgenomic RNA M of SARS-CoV-2

Marta Soszynska-Jozwiak[1], Agnieszka Ruszkowska[1], Ryszard, Kierzek[1], Collin A. O'Leary[2], Walter N. Moss [2], Elzbieta Kierzek[1*]

[1]Institute of Bioorganic Chemistry, Polish Academy of Sciences, Noskowskiego 12/14, 61-704 Poznan, Poland.

[2]Roy J. Carver Department of Biophysics, Biochemistry and Molecular Biology, Iowa State University, Ames, IA 50011, USA

*Corresponding authors:

Elzbieta Kierzek, Institute of Bioorganic Chemistry Polish Academy of Sciences, 61-704 Poznan, Noskowskiego 12/14, Poland, E-mail: elzbieta.kierzek@ibch.poznan.pl

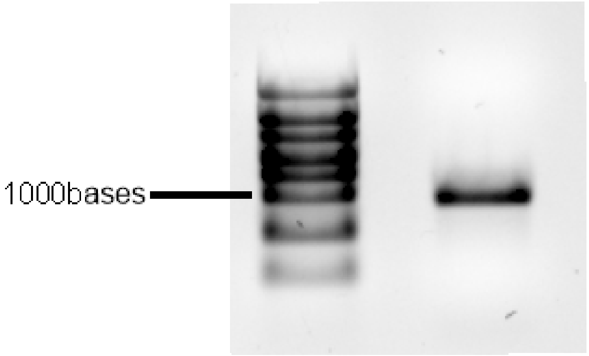

Figure S1. SgRNA M after folding by heating in 80°C for 5 min. in water and slowly cooled to 50°C. In 50°C buffer was added and slowly cooling to 37°C. Folding buffer: 300mM NaCl, 5mM $MgCl_2$, 50mM HEPES, pH 7.5.

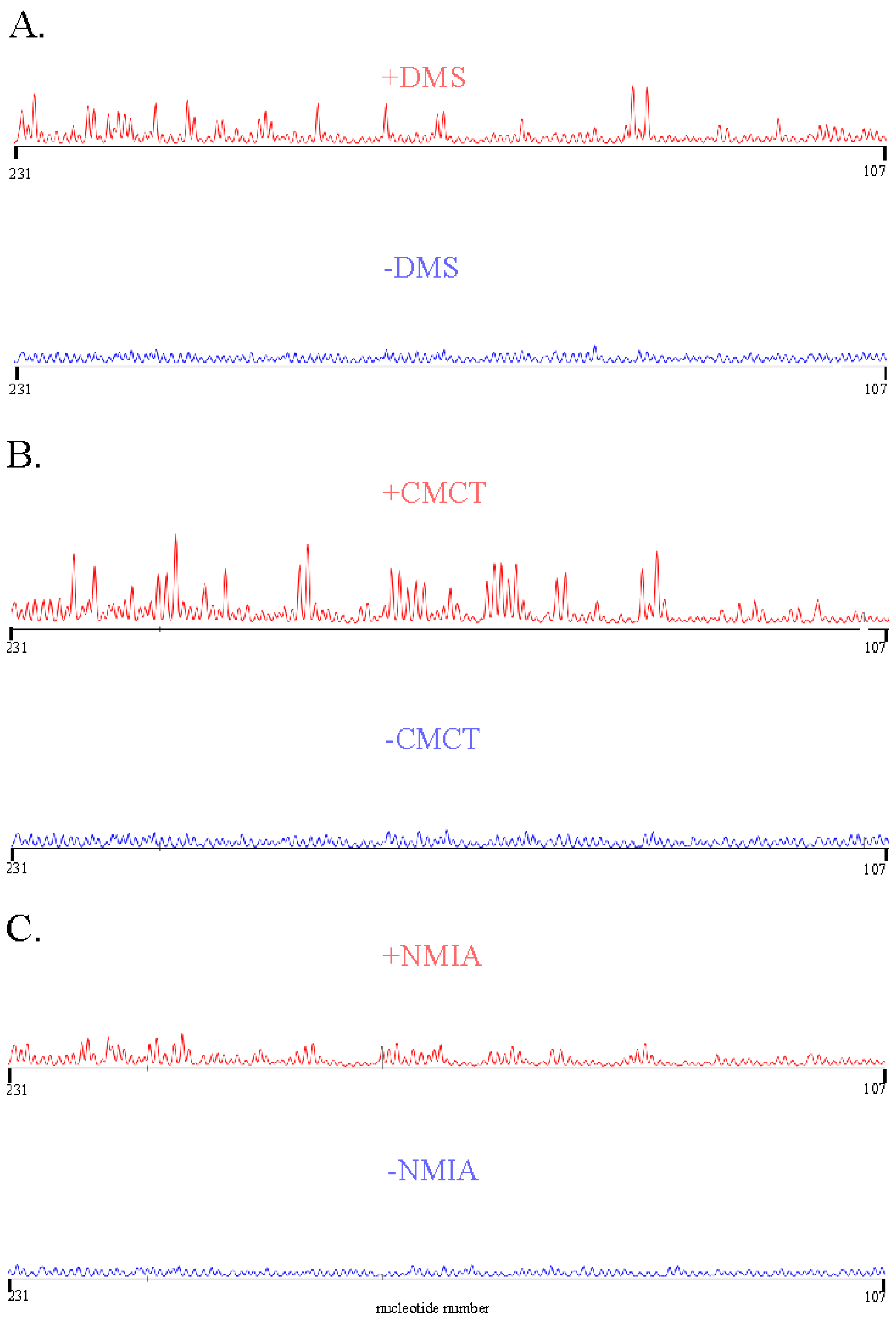

Figure S2. A-Example of capillary electrophoresis raw data for nucleotides 107-231 showing CMCT modified RNA (red line), unmodified control (blue line), B- Example of capillary electrophoresis raw data for nucleotides 107-231 showing DMS modified RNA (red line), unmodified control (blue line), C-Example of capillary electrophoresis raw data for nucleotides 107-231 showing NMIA modified RNA (red line), unmodified control (blue line).

NMIA mapping of sgRNA M

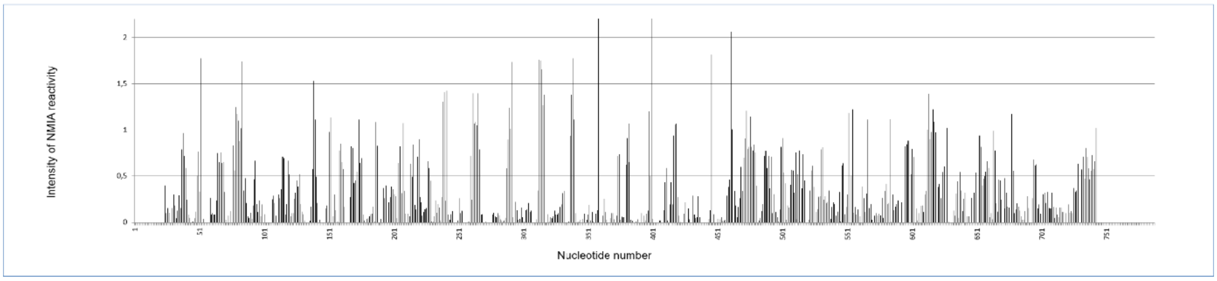

DMS mapping of sgRNA M

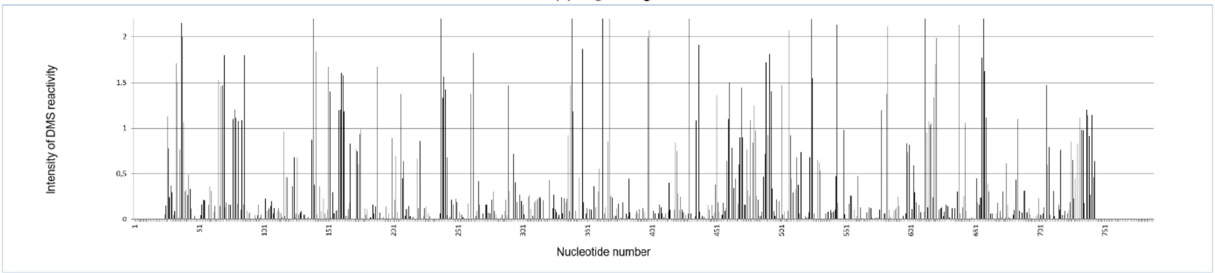

CMCT mapping of sgRNA M

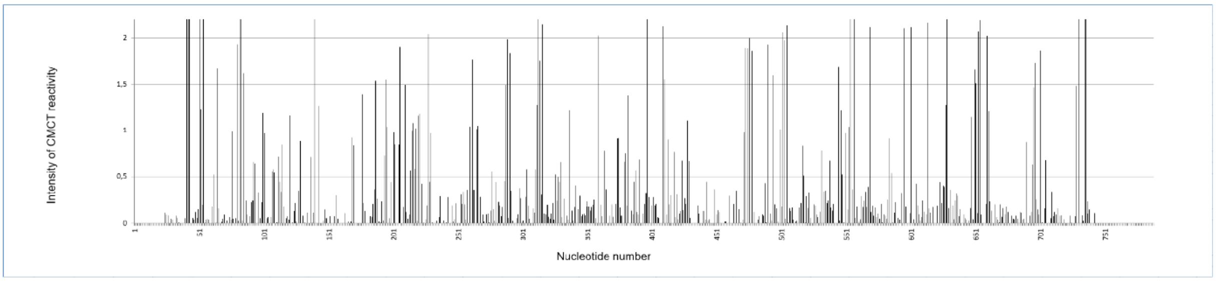

Figure S3. sgRNA M nucleotides reactivity diagrams. The sgRNA M chemical mapping experiments were performed at 37 °C with NMIA, DMS and CMCT.
